# Exploring factors influencing chiropractors’ adherence to radiographic guidelines for low back pain using the Theoretical Domains Framework

**DOI:** 10.1186/s12998-022-00433-5

**Published:** 2022-05-09

**Authors:** Daphne To, Amanda Hall, André Bussières, Simon D. French, Rebecca Lawrence, Andrea Pike, Andrea M. Patey, Debbie Brake-Patten, Lino O’Keefe, Barbara Elliott, Diana De Carvalho

**Affiliations:** 1 Division of Community Health and Humanities, Faculty of Medicine, Memorial University of Newfoundland, St. John’s, NL A1B 3V6 Canada; 2 Primary Healthcare Research Unit, Faculty of Medicine, Memorial University of Newfoundland, St. John’s, NL Canada; 3 School of Physical and Occupational Therapy, Faculty of Medicine and Health Sciences, McGill University, Montreal, QC Canada; 4 Département chiropratique, Université du Québec à Trois-Rivières, Trois-Rivières, QC Canada; 5 Department of Chiropractic, Macquarie University, Sydney, NSW Australia; 6 Centre for Implementation Research, Ottawa Hospital Research Institute – General Campus, Ottawa, ON Canada; 7 Private Practice, Stephenville, NL Canada; 8 Private Practice, Bay Roberts, NL Canada; 9 Patient Engagement Partner, North Bay, ON Canada; 10 Faculty of Education and Professional Studies – School of Nursing, Nipissing University, North Bay, ON Canada

**Keywords:** Diagnostic imaging, Radiography, Chiropractors, Low back pain, Guidelines, Theoretical Domains Framework, Barriers and enablers

## Abstract

**Background:**

The inappropriate use of lumbar spine imaging remains common in primary care despite recommendations from evidence-based clinical practice guidelines to avoid imaging in the absence of red flags. This study aimed to explore factors influencing ordering behaviours and adherence to radiographic guidelines for low back pain (LBP) in chiropractors in Newfoundland and Labrador (NL), Canada.

**Methods:**

We conducted two focus groups in December 2018 with chiropractors in different regions of NL (eastern, n = 8; western, n = 4). An interview guide based on the Theoretical Domains Framework (TDF) served to identify perceived barriers to, and enablers of, target behaviours of guideline adherence and managing LBP without X-rays. We conducted thematic analysis of chiropractors’ statements into relevant theoretical domains, followed by grouping of similar statements into specific beliefs. Domains key to changing radiographic guideline adherence, LBP imaging behaviours, and/or informing intervention design were identified by noting conflicting beliefs and their reported influence on the target behaviours.

**Results:**

Six of the 14 TDF domains were perceived to be important for adherence to radiographic guidelines and managing non-specific LBP without imaging. Participating chiropractors reported varying levels of knowledge and awareness of guidelines for LBP imaging (*Knowledge*). Many chiropractors based their decision for imaging on clinical presentation, but some relied on “gut feeling” (*Memory, attention, and decision processes*). While chiropractors thought it was their role to manage LBP without imaging, others believed ordering imaging was the responsibility of other healthcare providers (*Social/professional role and identity*). Contrasting views were found regarding the negative consequences of imaging or not imaging LBP patients (*Beliefs about consequences*). Communication was identified as a skill required to manage LBP without imaging (*Skills*) and a strategy to enable appropriate imaging ordering behaviours (*Behavioural regulation*). Chiropractors suggested that access to patients’ previous imaging and a system that facilitated better interprofessional communication would likely improve their LBP imaging behaviours (*Behavioural regulation*).

**Conclusion:**

We identified potential influences, in six theoretical domains, on participating chiropractors’ LBP imaging behaviours and adherence to radiographic guidelines. These beliefs may be targets for theory-informed behaviour change interventions aimed at improving these target behaviours for chiropractors in NL.

**Supplementary Information:**

The online version contains supplementary material available at 10.1186/s12998-022-00433-5.

## Introduction

Low back pain (LBP) is experienced by many people worldwide and is a large social and economic burden [1, 2]. Most LBP is considered to be non-specific, with no pathoanatomical cause of pain; as such, diagnostic imaging has limited utility for assessment and management in this population [3]. Imaging for LBP may be considered appropriate when there is clinical suspicion of serious pathology (e.g., tumour, infection, fracture) that would alter the management of non-specific LBP [3]. However, serious pathology is estimated to be the cause of LBP in less than 5% of patients with LBP in primary care settings [3–5].

Clinical practice guidelines for the management of LBP recommend against the use of routine imaging, including lumbar radiography, for non-specific LBP [6, 7]. Routine imaging does not improve patient outcomes, increases exposure to unnecessary harms, and increases costs to the healthcare system [8–11]. Guidelines specific to diagnostic imaging in both the fields of medicine [10] and chiropractic [12] recommend delaying imaging for 4–6 weeks in the absence of red flags. Despite this, utilisation of imaging outside of guideline recommendations for the management of LBP remains common in both primary and emergency care settings [13, 14]. A recent systematic review and meta-analysis by Jenkins and colleagues [13] found that in patients referred for imaging in primary care settings, approximately 32–35% of patients had an inappropriate referral, while in patients presenting for care, approximately 7–28% of patients had an inappropriate referral. A recent study by our team [15] surveyed chiropractors in the Canadian province of Newfoundland and Labrador (NL) about their knowledge and adherence to radiographic guidelines for LBP and identified that half of the respondents were unaware of, or did not know, current guideline recommendations. In the same study, adherence to guidelines measured using clinical vignettes ranged from 38 to 88% for not ordering an X-ray when it was not indicated [15]. Thus, there appears to be a need to explore factors influencing the adherence to radiographic guidelines for the management of LBP by chiropractors in NL.

Psychological theory can be used to understand the behaviour of healthcare providers, as well as to develop interventions aimed at behaviour change [16]. The Theoretical Domains Framework (TDF) maps 128 theoretical constructs into 12 domains and was initially developed by a group of international health psychology theorists, health services researchers, and health psychologists to understand the behaviours of healthcare providers [17]. The original TDF was revised and validated to include 14 domains covering 84 theoretical constructs (Table 1) [18, 19].

**Table 1:** Domains from the TDF and their descriptions adapted from Atkins et al. [19]

Theoretical domain	Definitions
Knowledge	An awareness of the existence of something
Skills	An ability or proficiency acquired through practice
Social/professional role and identity	A coherent set of behaviours and displayed personal qualities of an individual in a social or work setting
Beliefs about capabilities	Acceptance of the truth, reality or validity about an ability, talent or facility that a person can put to constructive use
Optimism	The confidence that things will happen for the best or that desired goals will be attained
Beliefs about consequences	Acceptance of the truth, reality, or validity about outcomes of a behaviour in a given situation
Reinforcement	Increasing the probability of a response by arranging a dependent relationship, or contingency, between the response and a given stimulus
Intentions	A conscious decision to perform a behaviour or a resolve to act in a certain way
Goals	Mental representations of outcomes or end states that an individual wants to achieve
Memory, attention, and decision processes	The ability to retain information, focus selectively on aspects of the environment and choose between two or more alternatives
Environmental context and resources	Any circumstance of a person’s situation or environment that discourages or encourages the development of skills and abilities, independence, social competence and adaptive behaviour
Social influences	Those interpersonal processes that can cause individuals to change their thoughts, feelings, or behaviours
Emotion	A complex reaction pattern, involving experiential, behavioural, and physiological elements, by which the individual attempts to deal with a personally significant matter or event
Behavioural regulation	Anything aimed at managing or changing objectively observed or measured actions

The TDF has been used widely to explore barriers and enablers to implementing evidence-based practices in various health disciplines [19, 20]. The TDF has also been used among chiropractors to identify factors likely to influence adherence to diagnostic imaging guidelines for spine disorders [21]. Bussières and colleagues used focus groups to explore chiropractors’ views about managing LBP without imaging in two states in the United States as well as in two provinces in central Canada [21]; however chiropractors in NL, a Canadian province where the utilisation rate of radiography for LBP by chiropractors is unknown, were not included. Informal data tracked by the Newfoundland and Labrador Chiropractic Board suggests a utilisation rate of up to 36% (based on data from the Avalon Peninsula for 2015, assuming two new patients per week).

The purpose of this study was to use the TDF to explore perceived barriers to, and enablers of, LBP radiographic guideline adherence and managing LBP without X-rays among chiropractors in NL, Canada. Findings may inform intervention design that enhances adherence to radiographic guidelines and managing LBP without X-rays.

## Methods

### Design

This was a qualitative study conducted using focus groups with chiropractors in the province of NL. Focus groups were chosen as a data collection method because they are useful in exploring people’s knowledge, experiences, and attitudes towards a given topic with the use of group interaction [22]. Group interaction was important for this study to explore the degree of consensus on the participating chiropractors’ perceived barriers to and enablers of LBP radiographic guideline adherence and managing LBP without X-rays. This study was reported according to the COnsolidated criteria for REporting Qualitative research (COREQ) checklist [23].

### Participants

All 69 chiropractors registered with the Newfoundland and Labrador Chiropractic Association (NLCA) were invited by email to participate in the focus groups. Chiropractors who were interested in participating were asked to provide their name and contact information to the research team. All chiropractors who wanted to participate and met the inclusion criteria (i.e., currently practising in NL) were included in the study.

### Interview topic guide

The target behaviours of interest were “adherence to radiographic guidelines for LBP” and “managing non-specific LBP without lumbar spine X-rays”. An interview topic guide informed by the original version of the TDF [17] was used (Additional File 1). The interview guide was adapted from one used in a previous study by Bussières and colleagues exploring chiropractors’ beliefs about the management of LBP without imaging [21]. Face and content validity of the previous interview guide were assessed by experts in knowledge translation and chiropractors [21]. The current interview guide contained a fewer number of questions compared to the previous one used by Bussières and colleagues [21] due to feasibility (i.e., time limitations), as well as some revised and reworded questions for clarity. Prompts were used when necessary for further clarification or to allow for participants to elaborate on previous responses. The number of questions ranged between one to five for 11 domains, for a total of 26 questions. No questions were specifically asked for the domain *Behavioural regulation*; however, prompts were asked in relation to this domain.

### Procedure

Two focus group interviews of four to eight chiropractors were held between June and December 2018. The interviews were conducted by experienced female interviewers (AH and/or AP) employed as academic faculty and/or researchers with an interest in primary care and LBP research. The interviewers were trained in qualitative methods and interview techniques, with over 15 years of experience. Participants learned about the interviewers at the start of the focus group via verbal introductions. Participants learned about the intentions and objectives of the focus group through the study information letter. There was no relationship between interviewers and participants established prior to the start of the study. Interviews lasted between 50 and 75 min and were digitally recorded. Field notes were taken in case of audio recording failure by a non-participant observer during one of the focus groups (RL), while no field notes could be taken for the other focus group due to limitations in availability of research personnel. As there was no audio recording failure, field notes were not used for the data analysis. Data were transcribed verbatim and de-identified prior to analysis. No repeat interviews were carried out. Transcripts were not returned to participants for comments and/or corrections and participant checking of findings was not performed.

### Analysis

#### Stage 1: Coding into theoretical domains

Two researchers trained in the TDF (DT, RL) independently coded participants’ responses into the relevant theoretical domain(s), guided by their understanding of the constructs within a domain, using NVivo (Release 1.0, QSR International, Melbourne, Australia). The revised TDF with 14 domains was used to code and analyse the data [18, 19]. For instances where an utterance was highly relevant to more than one domain, it was coded into multiple domains. Utterances unlikely to be relevant to lumbar spine X-ray ordering practices or radiographic guideline adherence were not coded. Any disagreements in coding of the domains by the independent researchers were discussed to reach consensus. Participant quotations were not presented in the manuscript in order to preserve anonymity due to the small sample size and small number of chiropractors eligible for the study.

#### Stage 2: Identifying specific beliefs and overarching themes

One researcher (DT) independently generated statements representing specific beliefs (i.e., belief statements) from each utterance that captured the core thought. A specific belief is a statement that provides greater detail about the role of the domain in influencing the behaviour [24]. Specific beliefs are meant to capture a meaning that was common to multiple utterances by the participants. Utterances that were considered similar to a previously identified utterance were coded as two instances of the same belief. Specific beliefs representing the same theme were grouped together. The specific belief statements were reviewed by a second researcher (RL) and consensus was achieved through discussion. Overarching themes were proposed for each domain by one researcher (DT) and reviewed by another (RL) for consensus.

#### Stage 3: Identifying relevant domains

Relevant and non-relevant domains were identified by one researcher (DT) and independently reviewed by two researchers (RL and AMP). Domains were considered relevant if they were potentially important for changing (i.e., increasing or decreasing) radiographic guideline adherence or LBP imaging behaviours or were important for informing the design of an intervention to improve these target behaviours. When identifying relevant domains, two factors were considered concurrently: the presence of conflicting beliefs and the perceived strength of the beliefs that may affect the target behaviours [19]. A frequency count of beliefs was not warranted for determining relevant domains, as focus group interviews may not have captured nonverbal behaviours such as nodding [19].

### Ethics

Ethics approval was obtained from the Newfoundland and Labrador Health Research Ethics Authority (#2017.292) prior to the start of this study. Participants read an information letter prior to the focus group interview and provided written consent to participate.

## Results

### Participants

Two focus groups were conducted with a total of 12 chiropractors: one focus group in St. John’s, NL in June 2018 (n = 8; 3 female, 5 male) and one in Corner Brook, NL in December 2018 (n = 4; 2 female, 2 male). Years in practice ranged from less than 5 years to over 30 years. No participants withdrew from the study. In the province of NL, all chiropractors are able to order X-rays through their Regional Health Authority.

### Key themes identified within relevant domains

Six domains (of the 14) were identified as potentially important for changing radiographic guideline adherence and LBP imaging behaviours: *Knowledge*; *Skills*; *Social/professional role and identity; Beliefs about consequences; Memory, attention, and decision processes; and Behavioural regulation*. See Table 2 for the overarching themes and belief statements for the relevant theoretical domains. The number of overarching themes for each relevant domain ranged from two to four.

**Table 2:** Summary of theoretical domains identified from two focus groups (n = 12) as key to adherence to LBP radiography guidelines and LBP imaging ordering behaviours (including overarching themes and belief statements)

Domain	Overarching theme	Example belief statements
Knowledge	Knowledge and awareness of LBP radiography guidelines and indications for imaging. (Enabler)	I know the limitations of X-rays and when it would be appropriate to choose X-ray as an imaging modality
		I am aware of guidelines and/or indications for LBP and imaging
		I agree with the content of the guidelines for imaging and LBP
		I think the guidelines are evidence-based
		My knowledge of indications for imaging comes from school
	Lack of awareness and/or knowledge of LBP radiography guidelines. (Barrier)	I do not follow a guideline
		I have limited knowledge/awareness of guidelines for imaging
Skills	Adequate training is required to manage LBP without imaging. (Enabler)	A lot of expertise and training is needed in order to manage someone with LBP (and determine if they need an X-ray)
	Having good communication skills is important for managing LBP without imaging. (Enabler)	Good communication skills are required for managing LBP without X-rays
Social/professional role and identity	Chiropractors’ responsibility to manage LBP without imaging. (Enabler)	It is my responsibility as a clinician to manage someone’s LBP without taking an X-ray
		Chiropractors should not be routinely taking X-rays
	Other healthcare providers’ responsibility to manage LBP without imaging. (Barrier)	It is the medical doctor’s role to order imaging
Beliefs about consequences	Negative consequences to imaging for LBP. (Enabler)	Radiation is a negative consequence of taking X-rays
		Cost to the healthcare system is a negative consequence of taking X-rays
		Delayed treatment (waiting for results) is a negative consequence of taking X-rays
		Exposure to infectious diseases is a negative consequence of sending a patient for an X-ray
		Patient worry is a negative consequence of taking an X-ray
	Negative consequences to not using imaging for LBP. (Barrier)	Missing a diagnosis is a potential negative consequence of NOT taking an X-ray
	Neutral consequences to not using imaging for LBP. (Enabler)	The plan of management does not change with taking an X-ray
Memory, attention, and decision processes	Decision for LBP imaging is based on a patient’s clinical presentation. (Enabler)	I decide whether a patient needs an X-ray based on their clinical presentation
	Decision for LBP imaging is based on gut feeling. (Barrier)	I would decide to order an X-ray (instead of following the guidelines) if I have a gut feeling that there is something else going on
	Able to remember indications for LBP imaging. (Enabler)	I can remember indications for when a patient needs imaging/needs a referral
Behavioural regulation	Communication is a strategy that can be used to reduce imaging for LBP. (Enabler)	I manage LBP without X-rays by explaining to my patients why they do not need X-rays
	Continuing education requirements is a strategy that can be used to reduce imaging for LBP. (Enabler)	Continuing education in radiology helps me manage LBP [with or without X-rays]
	Having access to a patient’s previous imaging is a strategy that can be used to reduce imaging for LBP. (Enabler)	Being able to access previous X-rays/reports helps me manage LBP without taking an X-ray
	A better health system organisation that facilitates better communication amongst health care professionals would help with reducing imaging for LBP. (Enabler)	Having a system to easily communicate with physicians and access previous imaging would help me better manage LBP [without X-rays]

*LBP* low back pain

The participating chiropractors reported conflicting levels of knowledge, awareness, and utilisation of radiographic guidelines for LBP (domain: *Knowledge*). Some chiropractors were aware of, and remembered, guidelines and indications for the appropriate use of imaging for LBP. They also reported making their decisions for LBP imaging based on whether a patient’s clinical presentation was in line with the indications from these guidelines (*Knowledge*; *Memory, attention, and decision processes*). However, some chiropractors did not use or were unable to identify specific guidelines and their recommendations (*Knowledge*). Instead, they reported that their decisions for LBP imaging were sometimes based on their “gut feeling” (*Memory, attention, and decision processes*). Additionally, most chiropractors interviewed reported relying on their training as chiropractic students and with continuing education courses to develop their knowledge of guideline recommendations (*Knowledge*).

Most chiropractors interviewed were aware of negative consequences related to the use of imaging for LBP when it was not indicated. These included increased radiation to patients, potential for delayed treatment, negative psychological impact on patients, and increased cost to the healthcare system. Participating chiropractors also reported that imaging for LBP may not change the plan of management for a patient (neutral consequence). However, some chiropractors also reported that missing a diagnosis is a potential negative consequence to not using imaging for LBP (*Beliefs about consequences*).

Most chiropractors interviewed believed it was their responsibility and job to manage people with LBP without imaging and they shared the belief that X-rays should not be routinely used in chiropractic practices. Despite this, a few chiropractors felt it should also be the role of other healthcare providers to manage LBP without imaging, particularly since many patients have seen other healthcare providers prior to the chiropractor (*Social/professional role and identity*).

All chiropractors agreed that communication was a skill that was required to manage LBP without imaging (*Skills*), as well as a strategy they often used to reduce the use of imaging for LBP in their practices (*Behavioural regulation*). When asked about other ways to improve guideline adherence and imaging ordering behaviours, most chiropractors interviewed suggested strategies at the individual and system levels. They suggested that continuing education requirements have been, and continue to be, a helpful strategy for reducing their use of imaging for LBP (*Behavioural regulation*). At a system level, chiropractors believed that having access to patients’ previous imaging could be a strategy to reduce future imaging for LBP (*Behavioural regulation*). Most chiropractors interviewed noted that while they were often able to request to view patients’ previous imaging, a health system that facilitates better communication among healthcare professionals would make access to previous imaging easier as well as help to reduce additional imaging ordering for LBP (*Behavioural regulation*).

### Domains less likely to inform intervention design to change target behaviours

Eight domains from the TDF (*Beliefs about capabilities; Optimism; Reinforcement; Intentions; Goals; Environmental context and resources; Social influences; and Emotion*) appeared to be less relevant as factors influencing participant chiropractors’ adherence to radiographic guidelines for LBP and therefore less likely to inform intervention design to improve these target behaviours (Table 3). The number of overarching themes for each domain that was less relevant ranged from zero to three. Participating chiropractors felt confident in their ability to manage LBP without imaging (*Beliefs about capabilities*). Their imaging ordering behaviours were not influenced by their previous experiences (e.g., managing patients with back pain from pathology) (*Reinforcement*), nor were they influenced by their patients or colleagues (*Social influences*). They wanted to manage LBP without imaging (*Intentions*) and viewed imaging as low priority during their initial assessment of patients with LBP (*Goals*). They reported that their decisions for ordering imaging were not due to fear or worry of missing pathology (*Emotion*). The chiropractors noted many existing resources that were available to them, including increased time and having access to patients’ previous imaging, which allowed them to manage LBP without imaging (*Environmental context and resources*). No utterances were coded under the domain of *Optimism*.

**Table 3:** Summary of theoretical domains identified from two focus groups (n = 12) as not relevant to adherence to LBP radiography guidelines and LBP imaging ordering behaviours (including overarching themes and belief statements)

Domain	Overarching theme	Example belief statements
Beliefs about capabilities	Confidence and control of LBP imaging ordering behaviours. (Enabler)	I am confident that I can manage LBP without X-rays
		I feel that I have control over the decision to manage LBP with or without X-rays
		It is easy for me to decide if I should order an X-ray or not
	Lack of confidence in the patient’s case presentation. (Barrier)	When I am not confident, I am more likely to refer for X-rays
Optimism	None identified	None
Reinforcement	Previous clinical experiences help to reduce use of imaging for LBP. (Enabler)	My previous experiences with diagnosing pathology does not influence my decision to always use X-ray with future patients with LBP
		My previous experiences with patients not needing X-ray influences my decision to NOT always use an X-ray for patients with LBP
		My previous clinical experiences help with deciding whether or not a patient requires an X-ray
Intentions	Positive intentions to managing LBP without imaging. (Enabler)	I want to manage patients with LBP without imaging
Goals	Imaging is low priority for management of patients with LBP. (Enabler)	Taking X-rays is low priority compared to taking a history and conducting a physical examination
Environmental context and resources	Many available resources (e.g., time, patient’s previous imaging) to help manage LBP without imaging. (Enabler)	Most patients have had X-rays that I can have access to
		My decision to take an X-ray is not influenced by time constraints
		There are clinical resources available to help manage LBP without X-rays
Social influences	Not influenced by others’ views on imaging for LBP. (Enabler)	I am not influenced by patients wanting X-rays for their LBP
		My decision to take an X-ray is not influenced by my colleagues
		I am not influenced by campaigns supporting reducing unnecessary imaging (and would still take an X-ray if necessary)
		My decision to take an X-ray is not influenced by patients in lots of pain
Emotion	Not influenced by fear or worry. (Enabler)	Fear does not influence my decision to take an X-ray
		Not using imaging does not make me worried that I missed a diagnosis
	Feel comforted with having ability to order X-rays if needed. (Barrier)	It gives me comfort knowing that I can take an X-ray if I need to

*LBP* low back pain

## Discussion

This study applied the TDF to help understand factors that influenced chiropractors’ adherence to radiographic guidelines for LBP and their imaging ordering behaviours. Results from focus groups with chiropractors in NL showed that the main factors influencing their adherence to radiographic guidelines and LBP imaging ordering behaviours could be coded into six key domains: *Knowledge*; *Skills*; *Social/professional role and identity; Beliefs about consequences; Memory, attention, and decision processes; and Behavioural regulation*. Many of these factors were enablers to guideline adherence and potentially reduced the ordering of non-indicated imaging for LBP.

In our current study, there were varied levels of knowledge and awareness of radiographic guidelines, with some chiropractors being able to state specific guidelines, and others not being aware of guidelines at all (*Knowledge*). This aligns with a similar qualitative study conducted by Bussières and colleagues [21] in a population of chiropractors in North America. This also aligns with a previous cross-sectional survey of chiropractors in NL, which found that although half of respondents were aware of radiographic guidelines, one quarter of them did not use guidelines to inform clinical decisions [15]. While chiropractors may have knowledge of the guidelines, their level of understanding of these guidelines was not explored. This may account for chiropractors expressing that their decision for ordering imaging was sometimes due to their “gut feeling” (*Memory, attention, and decision processes*) or because not ordering imaging may result in a missed diagnosis (*Beliefs about consequences*). Not having a deeper understanding of the information contained in radiographic guidelines may result in diagnostic uncertainty, which has also been identified as a barrier to reducing general medical practitioner referral for non-indicated imaging in a study by Jenkins and colleagues [25].

Many enablers to guideline adherence and appropriate imaging ordering behaviours for LBP found in our current study were similar to those previously reported in the literature. Compared to other chiropractors in North America, participating chiropractors in NL also believed that it was their role to manage LBP without imaging and that chiropractors should not routinely use imaging in their practices (*Social/professional role and identity*) [21]. Similarly, compared to known enablers to reducing general medical practitioner referral for non-indicated imaging, participating chiropractors in NL also reported awareness of guidelines (*Knowledge*), being able to recall guidelines (*Memory, attention, and decision processes*), and knew the potential negative consequences of non-indicated imaging (*Beliefs about consequences*) [25]. Additionally, having good communication skills were seen both as an important skill to help with managing LBP without imaging (*Skills*) and as a potential strategy to use for interventions targeting non-indicated imaging for LBP (*Behavioural regulation*) [25].

Our study identified eight theoretical domains that appeared to be less relevant as factors influencing chiropractors’ adherence to radiographic guidelines for LBP. Most of the beliefs within these theoretical domains represented enablers or had no influence to these target behaviours and were therefore less likely to inform intervention design. This is different from what was previously reported in the literature on factors affecting guideline adherence and appropriate imaging ordering behaviours in chiropractors and physicians (i.e., they were previously identified as barriers in other populations) [21, 25, 26]. The chiropractors interviewed in our study reported that their imaging ordering behaviours were not influenced by patients, colleagues, or other healthcare providers (*Social influences*), whereas chiropractors and physicians in previous studies stated they were influenced by their patients wanting to receive an X-ray [21, 26]. The results of our current study align with the results of a survey of chiropractors from NL, where 87% of chiropractors reported that they were not likely to refer for an X-ray just because patients expected it [15]. Similarly, chiropractors in our study felt confident in their ability to manage LBP without imaging, while other studies have demonstrated uncertainty and a lack of confidence (*Beliefs about capabilities*) [21, 25]. When compared to other studies on physician-reported barriers to radiographic guideline adherence, chiropractors in our study reported that time was not a limiting factor in their ability to manage LBP without imaging (*Environmental context and resources*) [25, 26]. This may be due to differences in the organisation of a typical clinical encounter between chiropractors and physicians, such as appointment duration and/or number of conditions discussed per appointment. However, it may also be due to other factors such as continued messaging around this issue, years of experience, etc. These interprofessional factors may need to be taken into consideration when designing future interventions to target LBP radiographic guideline adherence.

### Strengths and limitations

A strength of our study is that we used the TDF to identify barriers and enablers to the adherence to radiographic guidelines for LBP, which may be used to inform the design of behaviour change interventions targeted at reducing non-indicated imaging for LBP [19]. There are several limitations to our study. Only two focus groups were conducted, resulting in the possibility that data saturation was not reached; however, there were many similar beliefs expressed between the two focus groups. A total of 12 chiropractors were included in the study, which represented approximately 17% of the chiropractors in NL. Since the chiropractors who participated in the study volunteered for the study themselves, it is possible that chiropractors who adhere to radiographic guidelines and have appropriate imaging ordering behaviours were more likely to volunteer for the study. Anecdotally, it has been suggested that chiropractors in the province of NL generally have a strong sense of community and similar practice styles (Diversified technique). The results of our study may be less generalisable to chiropractors in other regions where access to imaging is different or to chiropractors with different practice styles. Another limitation is that the interview guide used in our study was based on the original version of the TDF with 12 domains, allowing comparison with findings from a previous qualitative study conducted among Quebec and Ontario chiropractors, but the data were coded according to the more updated and validated version of the TDF with 14 domains. The updated version of the TDF is similar in structure and content to the original version of the TDF. Two key differences between the two versions of the TDF are that the domains were expanded into *Beliefs about capabilities* and *Optimism* (previously only *Beliefs about capabilities*), and *Beliefs about consequences* and *Reinforcement* (previously only *Beliefs about consequences*). This likely explains why no utterances were coded into the domain of *Optimism*, and thus may have been considered a less relevant domain.

### Future directions

Based on the factors we identified as key to influencing chiropractors’ LBP imaging ordering behaviours and adherence to radiographic guidelines, the domains can be mapped to behaviour change techniques [27] in order to develop an intervention to target these behaviours for chiropractors in NL.

## Conclusion

This study used the TDF to explore factors influencing chiropractors’ LBP imaging ordering behaviours and adherence to radiographic guidelines in the province of NL. The clinical behaviour of these participating chiropractors appeared to be influenced by the six relevant TDF domains of: *Knowledge*; *Skills*; *Social/professional role and identity; Beliefs about consequences; Memory, attention, and decision processes; and Behavioural regulation*. In particular, the varied levels of knowledge and awareness of radiographic guidelines for LBP (*Knowledge*) and the use of “gut feeling” for decision making about the need for lumbar spine radiography (*Memory, attention, and decision processes*) are barriers which may need to be explored further. Interventions to improve adherence to radiographic guidelines and management of LBP without imaging should address the theory-informed barriers identified in this study.

## Supplementary Information

**Additional file 1.** Interview topic guide informed by the Theoretical Domains Framework.

## Data Availability

To protect participant anonymity, recordings generated during the current study are not publicly available. Deidentified transcripts used and/or analysed during the study are available from the corresponding author on reasonable request.
