# Supplementary material for: Exploring factors influencing chiropractors’ adherence to radiographic guidelines for low back pain using the Theoretical Domains Framework
Source: Chiropr Man Therap. 2022 May 9;30:23. doi: 10.1186/s12998-022-00433-5 (PMC9082849; doi:10.1186/s12998-022-00433-5)
Supplement: Supplementary file 1 — Additional file 1. Interview topic guide informed by the Theoretical Domains Framework. [file 12998_2022_433_MOESM1_ESM.pdf]

Focus Group Interview Topic Guide

**Adapted from Bussières and Grimshaw, 2016*

Let's begin. To start, I would ask that you please state your name slowly for the benefit of the person who will be transcribing this interview session. It would be helpful if you could please repeat your first name before commenting during the early part of our discussion for the same reason.

Now, I would like to ask you to tell me, in general terms, how you manage new patients with spinal disorders in your practice. By that, I mean your usual routine once the patient is with you in the examining room, questions you ask, the examination procedures you do, and if x-rays are part of your routine initial evaluation. (*Prompts: what do you feel you do well? What do you feel you could do better?)*

Thank you.

Now for the rest of the interview, I have a few more specific questions. Some of these may seem repetitive, but this is on purpose. These questions have been developed to help our research team identify which theory of human behaviour applies in this area. I may also ask for clarification during the interview using probes such as, "what do you mean", "would you explain that", "what were you thinking at the time", "take me through the experience", "what skills are required to do so?", "how and why do you use it?".

Please note that for the purpose of this interview, "non-specific" refers to patients with uncomplicated mechanical back pain that varies with time and activity with no neurological deficits, fractures or indicators of potentially serious pathologies (i.e. red flags).

Skills

1. How much expertise or experience do you think one needs to have to effectively perform a history and examination of low back pain, with the goal of ruling out suspicion of red flags?

Beliefs about capabilities

2. Do you practice using a range of techniques? What are they? Do some of these techniques require the use of x-rays to establish a treatment protocol? (*Prompt: postural and biomechanical analysis for particular techniques*)
3. How confident are you that you can manage non-specific low back pain without having x-rays? (*Prompt: how easy or difficult is, and what skills are required to do so?*)
4. What could help you overcome these problems/barriers? (*Prompt: additional training, communication techniques, continuing education, educational material, online information. Please elaborate on whether or not you think communication skills are important for managing patients without taking x-rays? Why is that?)*

Motivation and goals

5. Do you feel motivated to manage patients without referring for plain x-ray of their lumbar spine?
6. What do you feel is the importance (i.e. priority) of x-rays in the context of other tasks like history taking and examination or others?

Beliefs about consequences

7. What are the benefits of managing a patient with non-specific neck and back pain without using x-rays? (*Prompt: exposure to ionizing radiation and costs?*)
8. Do you feel there are potential harms or disadvantages in managing patients with non-specific low back pain without any x-rays? (*Prompts: screening to prevent rare possible complications associated with spinal manipulative therapy, accurate prognosis, patient preference and satisfaction, medico legal concerns*)

Nature of the behaviours

9. How complex or difficult is it to diagnose a patient's condition without taking x-rays? Why? (*Prompt: patient's compliance, lack of training, lack of communication skills*)
10. How frequently do you x-ray patients? (*Prompt: how much of your practice is on musculoskeletal conditions? Do you systematically screen your patients for the presence of red flags? Do you assess the patient's motivation for wanting/not wanting x-rays, and if so, how? What do you usually say to patients who ask to undergo x-rays but where you find it is not clinically warranted? Do you follow-up to monitor patient process after treating them without prior x-rays? What is the outcome usually?*)

Environmental context and resources

11. Does running late influence whether or not you will decide to manage non-specific low back pain patients without taking x-rays?
12. Are there any resources available to help you manage non-specific low back pain patients without taking x-rays? (information pamphlet or posters to inform patients about potential risks of ionizing radiation exposure?)

Social influences

13. Are there instances where you may consider consulting other people for their opinion regarding the need for spine x-rays? (*Prompt: peers, managers, other professional groups, patients*)
14. How does the views of other colleagues influence your decision to x-ray your patients

Stress/concerns

15. Does seeing acute low back pain patients in apparent distress likely influence your decision to x-ray patients?
16. Would the fear of missing a pathology influence your decision to take an x-ray?

Knowledge about the guidelines

17. How well do you think you understand the evidence surrounding optimal use of x-rays for non-specific low back pain?
18. Do you adhere to any specific guideline to help you make informed decisions about when to x-ray patients for low back pain? (*Prompt: how do you use it? What do you think of it?*)
19. Do you agree with the guidelines? (*Prompt: what alternative, if any, would help you conform to the guideline recommendations?*)
20. Are the guidelines representative of the evidence (quality, appropriateness)?
21. What kind of additional information would most likely influence you changing your clinical management of non-specific low back pain? (*Prompt: RCT's, systematic reviews, discussion with colleagues, conferences/seminars*)

Decision process

22. How easy or difficult is it to decide if a particular new patient needs an x-ray or not?

23. What rules of thumb do you use to reach a decision, if any? (*Prompt: red flags, decision rules, guidelines etc.*)

Social/professional role and identity

24. Do you think it is an appropriate part of your job to manage back pain patients without referring for plain x-ray of their lumbar spine?

Studies conducted in Canada by Bussières et al. (2016) found that chiropractors reported philosophical beliefs and scope of practice that could be categorized into six strata, from traditional views (i.e., empirist or conservative) to the other end of the continuum where one has a rationalists view (i.e., evidence-based practice).

25. On the continuum from traditionalist to rationalist, where do you situate yourself?
26. Are there any circumstances where you might consider placing yourself at a different place on the continuum?

That is all the questions I had for you today. Has this session prompted anything other thoughts about this topic that we haven't asked about?

Overall, what were your thoughts about the interview?

References:

Bussieres AE, Patey AM, Francis JJ, et al. Identifying factors likely to influence compliance with diagnostic imaging guideline recommendations for spine disorders among chiropractors in north america: A focus group study using the theoretical domains framework. Implement Sci. 2012;7:82.

Bussieres A, Al Zoubi F, Stuber K, French S, Boruff J, Corrigan J, et al. Evidence-based practice, research utilization, and knowledge translation in chiropractic: a scoping review. *BMC Complement Altern Med.* 2016;16(1):216.
